# A cross-over study comparing an online versus a paper 7-day food record: focus on total water intake data and participant’s perception of the records

**DOI:** 10.1007/s00394-015-0945-7

**Published:** 2015-06-12

**Authors:** B. Monnerie, L. G. Tavoularis, I. Guelinckx, P. Hebel, T. Boisvieux, A. Cousin, L. Le Bellego

**Affiliations:** Danone Research, RD 128, 91767 Palaiseau Cedex, France; CREDOC, Paris, France; MXS, Paris, France; GFK-ISL, Paris, France

**Keywords:** Total water intake, Beverages, Fluids, Dietary record, Hydration

## Abstract

**Purpose:**

To compare (1) fluid, food and nutrient intake obtained with a paper versus an online version of a 7-day food record and (2) user’s acceptability of both versions of the food record.

**Methods:**

A cross-over study was carried out in 2010 in France. A total of 246 participants aged 18–60 years reported their food and fluid intake using both versions of the 7-day food record, separated by a 7- to 14-day washout period. To help participants in estimating consumed portions, both versions of the food record were supported by a photographic booklet of standard portions and containers. At the end of the study protocol, participants completed a questionnaire designed to assess the acceptability of the two questionnaires.

**Results:**

The reported water intake of fluids was significantly higher with the online version compared with the paper version (respectively 1348 ± 36 and 1219 ± 34 mL/day, *p* < 0.0001). No difference was found between methods in terms of energy intake and the consumption of most food categories, macro- and micronutrients. Furthermore, 77 % of the participants preferred the online method to the paper version.

**Conclusions:**

Fluid intake, but not food intake, reported with the online 7-day food record was higher in comparison with the paper version. In addition, the online version was preferred by users. In population surveys, the online record is therefore a relevant alternative, and even a preferred alternative in the case of fluid intake, to the paper record.

## Introduction

Several methods are available to assess individual food and nutrient intake [1]. The accuracy of data collection, however, is often hampered by the acceptability of comprehensive methods that may be perceived by subjects as long and boring [2].

Accurate assessment of fluid intake presents additional problems since fluids are consumed regularly throughout the day and packaging and container (glasses, cans, bottles, carton) size varies greatly. As a result, fluid consumption is rarely reported accurately during dietary surveys. In food studies reporting fluid consumption, there is often a marked difference in intake [3, 4] and the most likely explanation for such discrepancies is the use of different methods of measurement. Further research is therefore needed to accurately measure fluid intake during dietary surveys [4].

Given the need to improve acceptability and reduce the time spent by subjects on dietary surveys, new technologies have been introduced. Since three out of four people in Europe will be using Internet by the end 2014 [5], electronic interfaces are highly relevant and increasingly common, especially for dietary recording in younger generations accustomed to web technologies [6]. A study comparing online and paper versions of a self-administered anthropometric questionnaire highlighted easier data management of the online version [7]. Indeed, data processing appears to be faster and cheaper since data entry by a third party is no longer required. Furthermore, printing, mailing and processing of paper questionnaires are avoided in web-based studies, which considerably impact expenses, paper waste and the environment. Moreover, in case of multicentre or international surveys such as the NutriNet-Santé study or HELENA study, online questionnaires have the potential to acquire in a homogenous way the same type of data with a relatively limited amount of resources [8, 9].

However, the method of recording data, online versus on paper, might influence the recording. Therefore, the primary objective of the present study was to compare fluid, food and nutrient data collected using an *online* 7-day dietary record versus a *paper* 7-day dietary record. The secondary objective was to compare the acceptability of the two versions of the record by the participants.

## Methods

### Study design

**Fig. 1:**
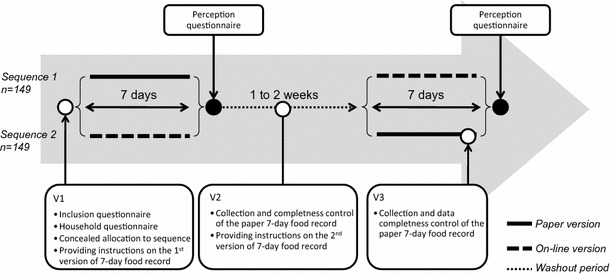
Summary of the study design

The study was designed as an observational, multicentre, cross-over survey. This cross-over (AB/BA design) study had two periods, one during which the paper version and one during which the online version of the 7-day food record was completed. Both periods were separated by a 7- to 14-day washout period. Participants were alternately upon recruitment allocated to one of the two sequences: sequence 1 started with completing the paper 7-day food record and sequence 2 with the online version. The study design is presented in Fig. 1.

Visit 1 (V1): participants were visited at their home by an interviewer who checked that the inclusion criteria were met. Participants meeting the inclusion criteria were informed about the study objectives and protocol. After signing an informed consent, short instructions were given on the first method of data collection to which they were randomly assigned to (i.e. paper or online version). If a participant was allocated to sequence 1, the paper 7-day food recorded was provided to the participant. If allocated to sequence 2, the participant started with the online 7-day food record. Participants were instructed to keep their usual dietary habits and not to change them through the full protocol. All participants completed a household questionnaire, reporting socio-economic data.

Visit 2 (V2): participants were visited a second time by the interviewer between the two periods. During this visit, instructions on the second method of data collection were given. The paper 7-day food record was also provided to participants of sequence 2. The interviewers checked with participants of sequence 1 the paper 7-day food record for completeness. All participants completed the perception questionnaire addressing the acceptability of the first version of the 7-day food record.

Visit 3 (V3): participants allocated to sequence 2 received a final visit from the interviewer at the end of the second period. During this visit, interviewers checked the paper 7-day food record for completeness. At the end of the second study period, all participants completed for a second time the perception questionnaire addressing this time the acceptability of the second version of the 7-day food record.

The study protocol was approved by the National Data Protection Authority (*Commission Nationale de l’Informatique et des Libertés*—*CNIL*) which oversees ethical issues and protection of individual data collection in France. All the procedures required to render individual data anonymous were performed. Written informed consent was obtained from each participant. However, owing to the descriptive and non-interventional design of this survey, approval by an ethics committee was not required, and the study was not submitted to registration.

### Participants and recruitment

The inclusion criteria of this study were as follows: having an age between 18 and 60 years, living in France, having a broadband Internet connection at home and being accustomed to using the Internet at least once a month. A quota system was devised to ensure that a broad, nationally representative cross section of households of different composition and type of areas (type/size of home town: rural/small town/medium-sized city/large city/Paris area) were recruited into the study. Participants were stratified according to sex, age (<40/≥40 years old) and professional activity (unemployed/lower socio-professional category/upper socio-professional category).

Interviewers approached individuals at random on the street or they went from door-to-door to recruit participants in order to reduce the selection bias. They continued this random recruitment until all quotas were achieved, and an equal balance between the aforementioned stratification groups was achieved.

### Dietary assessment

The 7-day food record used in this study has been used in large cohort surveys in the past, such as CCAF (*Comportements et Consommations Alimentaires en France* [10]) and INCA (*Enquête Individuelle et Nationale sur les Consommations Alimentaires* [3]). The paper 7-day food record was designed to be completed at home, and participants were asked to record all food and fluids consumed during and between meals over the week. It was supplied with a picture-based validated reference book from the SU.VI.MAX survey, which facilitated the assessment of portion size [11, 12].

The online version (MXS-Epidemio^®^, Paris, France) was based on the paper 7-day food record and was not used before in a large-scale nutritional survey neither on a period of 7 consecutive days. The software was only accessible online and was not available as an application for a smartphone. The software used a stepwise approach to guide the subject through the completion of the record, following the model of the multiple-pass approach used by the US Department of Agriculture [13]. In a first step, the type of food and fluid consumed at each moment of the day (breakfast, lunch, dinner or any solid or liquid food consumed between meals) was fully registered. In a second step, the portion sizes were entered. To help the subject in the portion size assessment, web screens with calibrated images from the same reference book [12] were shown. The online system performed a match with the corresponding model meal and questioned the user about any forgotten foods. These real-time control procedures helped to minimise the occurrence of missing data. The final validation of data collection was preceded by controls such as the number of food intakes registered, total energy consumption and calculated volume of water intake.

The nutritional content of foods and fluids was calculated based on French food composition tables [14]. *Total water intake* refers to water from food and fluids; *water from fluids* refers to the water content of the total drinking water (tap, bottled still, bottled sparkling) plus beverages of all kind (milk, coffee, soft drinks, alcohol, soup, etc.). *Fluid intake* is the total volume of drinking water and beverages of all kind.

### Perception questionnaire

Acceptability of the two different 7-day food records by the participants was assessed using an ad hoc online questionnaire designed to assess the user’s perception on the food records. The questionnaire consisted of 24 questions in total, of which five items were rated on a ten-point scale with a score of ten being the best or most favourable answer. Sixteen items were qualitative questions with a Likert type of scale of four graded answers (not at all, somewhat, rather, very). The questions covered several topics related to the assessment method (e.g. the introduction to the online or paper version, the user-friendliness and the overall impression of the 7-day food records). The mean time spent per day on recording their food and fluid intake was also reported in minutes by the participants.

### Criteria for between-methods comparison

To address the primary objective of the study, several criteria to compare the two version of the 7-day food record were defined in advance. The first criterion was the mean water intake from fluids per day (expressed in mL/day), and the second criterion was the mean total water intake (i.e. water from fluid and solid food intake) per day. The third criterion was the mean frequency of drinking acts, defined as the number of times per day any fluid type was consumed. The fourth group of criteria was the mean daily energy intake, the mean daily nutrient intake of lipids, carbohydrates (total, simple and complex), proteins, alcohol, fibre, calcium and vitamin D and the frequency of eating acts (one act accounted for each intake of food). The overall score of the perception questionnaire was also used to compare the methods.

### Statistical methods

The statistical model used to compare quantitative variables recorded with both versions of the 7-day record was an analysis of variance (ANOVA) with mixed factors (fixed and random factors). The order of sequence was included in the ANOVA model. With this model, the full analysis set (FAS) can be used. The perception questionnaire items was analysed using McNemar’s Chi-square test for paired samples. Data management and statistics were performed with SAS 9.2 software. The significance level was set at *p* < 0.05. A double-entry procedure was used for the paper-based record. If a dietary record was not completed for at least 4 days, it was considered as missing data.

## Results

### Study population

Overall, 298 participants were recruited into the survey. Among them, 246 participants (i.e. 82.6 %) completed the dietary assessment for at least 4 days for each method (FAS) and 243 participants completed 7 days for each method, i.e. 81.5 % of the global cohort (per protocol population set).

The cohort was composed of 59 % women. Participants were aged 18–60 years, with 53.5 % aged less than 40 years. More than 80 % used Internet almost every day before the study. There were no significant differences in the demographic characteristics of the participants of both sequences.

### Water from fluids and fluid intake

**Fig. 2:**
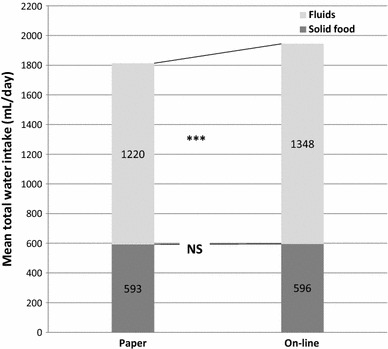
Mean daily water intake (mL/day) from fluid and solid food. Data are presented as mean ± SD and tested with ANOVA. ***Significantly different between paper and online methods (*p* < 0.0001); *NS* not significantly different

In the FAS sample, the mean total water intake calculated from data collected with the paper 7-day food record was significantly lower compared to the one from the online record (1812 and 1945 mL/day, respectively, *p* = 0.0005). This difference is due to the reporting of fluid intake: the mean daily water from fluid intake calculated from the paper-collected data was significantly lower (1220 mL/day) than the one from the online record (1348 mL/day; *p* = 0.0001). The volume of water derived from solid food did not depend on the method of data collection (Fig. 2). Among the different categories of fluids, the difference was statistically significant for still water, sparkling water, still soft drinks, milk and yoghurt drinks (Table 1).

**Table 1:** Total water intake and intake of different beverages recorded with the paper and online 7-day dietary record

	Paper (*n* = 228)	Online (*n* = 239)	*p* value*
Daily volume (mL) of water intake			
Total	1812 ± 40	1945 ± 42	*0.0005*
From fluids	1220 ± 34	1348 ± 36	<*0.0001*
Daily volume (mL)/beverage type			
Still water	532 ± 25	610 ± 29	*0.0044*
Sparkling water	44 ± 9	65 ± 11	*0.0005*
Hot drinks	376 ± 18	364 ± 17	0.3481
Alcoholic drinks	118 ± 16	105 ± 11	0.4106
Still soft drinks	95 ± 9	99 ± 10	0.5096
Sparkling soft drinks	78 ± 8	94 ± 11	*0.0391*
Dairy drinks	23 ± 5	42 ± 6	*0.0022*
Functional drinks	0.1 ± 0.1	1.4 ± 0.6	0.1219
Daily frequency of drinking acts	4.5 ± 0.1	5.0 ± 0.1	<*0.0001*

Values are presented as mean ± SD

*p* values less than 0.05 are in italics

* ANOVA with mixed factor with a *p* value cut-off of 0.05

The mean frequency of drinking acts was also significantly lower with the paper record than with the online version of the 7-day food record (Table 1).

### Food and nutrient intake

When comparing the intake of 42 food groups collected with the paper and online dietary record, the intake of only six food groups was significantly different. With the online dietary record, the mean daily intake was significantly higher for desserts (+23 %; *p* = 0.0138), pasta (+21 %; *p* = 0.0465), sweets (+13 %; *p* = 0.0222) and condiments (+21 %; *p* = 0.0096) and lower for bread and crackers (−10 %; *p* = 0.0231) and cooked or processed meat (−22 %; *p* = 0.0023) compared to the paper dietary record.

No significant difference was found for energy intake between the paper and the online versions (Table 2). The reported intake of all macro- and micronutrients was comparable between methods, except for simple carbohydrates, calcium and magnesium, which were significantly lower with the paper than with the online version, and for vitamin D that was higher with the paper version (Table 2).

**Table 2:** Energy and nutrient intake recorded with the paper and online 7-day dietary record

	Paper (*n* = 228)	Online (*n* = 239)	*p* value*
Daily energy intake (kcal)			
Total	1836 ± 41	1825 ± 39	0.7448
During the week	1769 ± 42	1763 ± 37	0.8695
During the week end	2005 ± 52	1983 ± 52	0.6913
Daily macronutrient intake (g)			
Total fat	73.8 ± 2.0	73.2 ± 1.7	0.7294
Carbohydrates	199 ± 5	202 ± 5	0.5464
Simple carbohydrates	*81.7* ± *2.4*	*87.3* ± *2.5*	*0.0033*
Proteins	77.1 ± 1.7	75.2 ± 1.5	0.1771
Alcohol	10.1 ± 1.2	9.8 ± 1.2	0.5620
Fibre	14.7 ± 0.4	14.9 ± 0.4	0.6217
Daily micronutrient intake			
Calcium (mg)	760 ± 20	802 ± 18	*0.0146*
Iron (mg)	11.3 ± 0.3	11.2 ± 0.2	0.7055
Magnesium (mg)	244 ± 5	253 ± 5	*0.0396*
Sodium (mg)	2641 ± 66	2698 ± 59	0.3477
Vitamin A (IU)	849 ± 41	932 ± 46	0.1108
Vitamin C (mg)	77.2 ± 3.3	79.8 ± 3.1	0.3745
Vitamin D (µg)	2.3 ± 0.1	2.0 ± 0.1	*0.0210*
Vitamin E (mg)	7.9 ± 0.3	8.0 ± 0.2	0.7666
Frequency of daily eating acts	10.7 ± 0.3	12.7 ± 0.3	<*0.0001*

Values are presented as mean ± SD

*p* values less than 0.05 are in italics

* ANOVA with mixed factor with a *p* value cut-off of 0.05

As for fluid intake, the reported frequency of eating acts was significantly lower with the paper record than with the online version (Table 2). The same trend was observed when considering the total intake (e.g. fluids and solid food together). The difference in frequency of eating and drinking acts between both versions of the record was driven by eating and drinking acts consumed at home (11.6 vs. 13.6 times/day for paper and online methods, respectively, *p* < 0.0001). The number of eating and drinking acts outside the home was similar.

### Perception assessment

**Fig. 3:**
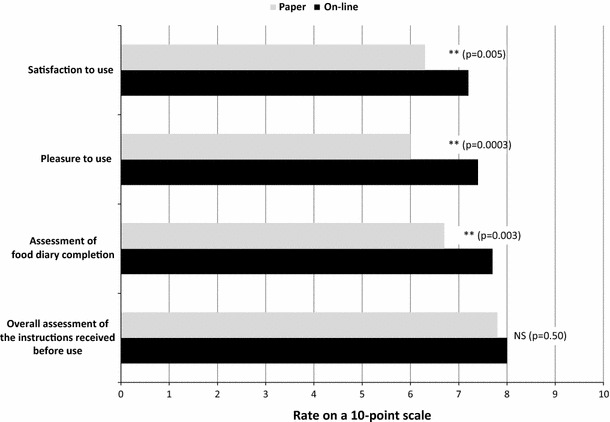
Mean acceptability scores based on the results of the perception questionnaire for the online and the paper 7-day dietary record. *NS* not significantly different. Participants rated each item from 0 (not at all) to 10 (totally)

A total of 100 participants (40.7 % of the FAS sample) completed the perception questionnaire twice, for example, after using each method of dietary data collection. On average, participants gave significantly higher scores to the online version regarding overall satisfaction with the tool, pleasure in using it and the feeling that the data recorded were accurate (Fig. 3). A general linear model indicated that the global perception score for the paper record did not significantly differ according to gender, sex, BMI, professional activity or frequency of Internet use (test Fisher 0.6512; *p* > 0.05). For the online record, only BMI but none of the other factors had a significant effect on the global perception score (test Fisher 0.048; *p* < 0.05). For both methods, the instructions received before starting data collection was perceived as satisfactory (score 7.9/10), useful (for 95 % of participants) and the information given to assess food intake was considered clear (for 95 % of participants). However, more participants considered the general layout and the graphical user interface as clear and friendly with the online version (Table 3). The online version was also assessed as more convenient, quicker and easier than the paper version (Table 3). Participants spent significantly more time completing the record using the paper version compared to the online version (34.4 vs. 28.5 min/day, *p* = 0.045). Participants also appeared to master the completion of the online record faster than the paper record (Table 3).

**Table 3:** Participants’ perception of the online and the paper version of the 7-day dietary record

	Paper (%)	Online (%)	*p* value
Appreciation of the general layout of the questionnaire			
Clear^a^	76.1	95.9	≤0.0001*
Friendly^a^	83.1	94.3	0.0018*
Perception of the data collection tool			
Convenient^a^	62.3	89.4	≤0.0001*
Quick^a^	51.6	85.2	≤0.0001*
Easy^a^	70.8	91.0	≤0.0001*
Number of days needed to master to the completion of the method			
From day 1^b^	20.0	34.4	0.0009*
From day 2^b^	37.7	48.4	
From day 3^b^	31.5	13.1	
Beyond day 4^b^	6.9	3.3	
Never^b^	3.8	0.8	

*p* values less than 0.05 are in italics

^a^Expressed as the percentage of participants who qualified the method “very” or “rather”

^b^Expressed as the percentage of participants who declared mastering the completion of the 7-day dietary record

* McNemar’s Chi-square test for paired sample

Finally, in response to the question “Which method did you prefer?”, 77.7 % of the participants who completed both methods preferred the online version, 13.2 % preferred the paper version, and 9.1 % did not report any preference.

## Discussion

The present study aimed to compare fluid, food and nutrient intake obtained with a paper versus an online version of a 7-day food record. To our knowledge, there is only one recently published pilot study that compare different dietary survey methods with a specific focus on the assessment of water from fluids [15]. This specific focus for fluid intake aimed to test our hypothesis that fluid intake is underestimated while using traditional paper food records. The results of the current study tend to support this hypothesis: total water intake, water from fluids and the reported volume of drinking water and other beverages types calculated from the online 7-day record were significantly higher compared to those calculated from the paper 7-day record. A possible explanation for these differences is the omission of drinking acts taking place outside meals. This explanation is derived from an observation made during a cross-sectional survey specifically assessing fluid intake where drinking acts took place not only during meals such as food intake, but also outside meal and this throughout the day [16]. The online interface was therefore designed to prevent fluids being forgotten during recording, especially between meals. An automatic reminder (contextual menu or pop-up window) appeared before entering each main meal, which was not available in the paper version. Indeed, the results suggest that the interactive interface did help participants to remember fluids consumed: the frequency of drinking acts declared with the online method was higher than with the paper record. On the other hand, in the absence of a referential method such as a biomarker, one cannot conclude that the data obtained with the online paper is more accurate. These automatic reminders prompting subjects to revise their reported intakes might lead to an over-reporting, in particular, for foods considered as healthy. Nevertheless, combining the observations of this cross-over trial and the cross-sectional survey discussed earlier, the assumption can be made that the online record more accurately reflects the actual fluid intake of participants. A practical key learning to retain from this finding is that whenever a participant is requested to record their food and fluid intake, extra questions regarding water and beverages intake within and outside meals should be raised.

As for the daily frequency of drinking acts, the daily frequency of eating acts was also higher with the online version of the record. Yet, the total daily energy intake and amounts of solid food were not statistically different between the two methods. These results are consistent with those obtained comparing online and paper versions of a multiple 24-h recall questionnaire [17]. Examining the intake of different food categories collected with the two versions of the dietary record, results showed no significantly difference for the 42 considered food categories, except for six groups. These differences in these few food categories are difficult to interpret and should be confirmed in future experiments that include biomarkers.

Another observation worth to comment relates to responders fatigue over the 7-day recording period. The statistical analysis tested the effect of the day of recording on the energy intake reported using a covariance model (data not shown), which could indicate the participant’s fatigue over time. With the online record, the effect of the day of recording is absent and energy intake remained constant over the week of reporting, whereas it decreased with the paper version. This observation suggests that using the online record reduces respondents fatigue over time.

These data are congruent with the evaluation of perception. Indeed, 44.6 % of the participants thought that they had not forgotten any food intake with the paper tool versus 57.4 % with the online tool. Conversely, 20.8 % of the participants using the paper tool felt that they had forgotten a lot of details during their recording versus 11.5 % with the online tool. These results provide support for the online tool in terms of the completeness of data recording. An addition argument in favour of the online tool is the fact that most participants declared that they preferred the online version of the questionnaire. This is consistent with another French study comparing a self-administered, online and paper-based socio-demographic and economic questionnaire. In all, 93.7 % of their participants preferred the online version. Substantial logistic and cost advantages were also demonstrated [8]. Against expectations, the perception scores for both tools were not different according to age, gender, professional activity or the frequency of Internet use. This lack of significant difference in perception score might, however, be due to the limited sample size of the stratified groups.

Despite the advantages listed above of an online questionnaire, the differential access to the Internet may raise concerns. Online questionnaires could exclude some populations such as elderly people and disadvantaged social classes [18]. However, Internet access is continually growing and may not be a real limitation in the near future [7]. In France, 64 % of homes subscribed to an Internet connection in 2011 versus 12 % in 2001 [19]. In the present study, the stratification of participants according to gender, age, socio-economic status and home environment aimed to prevent this selection bias.

To conclude, this study comparing two versions of a 7-day food questionnaire showed that an online record captured more drinking and eating acts than a paper version. Consequently, the online version resulted in a significantly higher mean intake of water from beverages and drinking water, despite the comparable or small differences between the two methods in terms of energy intake, and macro- and micronutrient intake. Even though a future confirmation with biomarkers is needed, these findings suggest that the online record more accurately reflects the actual daily fluid intake. Moreover, the online record was rated to be highly acceptable and user friendly and reduced the participants’ time spent on recording their intakes. These findings highlight the opportunity for the use of an online 7-day record as a medium to record fluid intake of large-scale populations.
